# Overexpression of cytochrome P450s in a lambda-cyhalothrin resistant population of *Apolygus lucorum* (Meyer-Dür)

**DOI:** 10.1371/journal.pone.0198671

**Published:** 2018-06-27

**Authors:** Congai Zhen, Yao Tan, Ling Miao, Jie Wu, Xiwu Gao

**Affiliations:** 1 Department of Entomology, China Agricultural University, Beijing, China; 2 Key Laboratory of Pollinating Insect Biology of the Ministry of Agriculture, Institute of Apicultural Research, Chinese Academy of Agricultural Science, Beijing, China; 3 Research Center for Grassland Entomology, Inner Mongolia Agricultural University, Hohhot, China; Institute of Zoology Chinese Academy of Sciences, CHINA

## Abstract

The mirid bug, *Apolygus lucorum* Meyer-Dür, has been an important pest of cotton crop in China, and is primarily controlled with insecticides, such as pyrethroids. To elucidate the potential resistant mechanisms of *A*. *lucorum* to lambda-cyhalothrin, a series of biological, biochemical, and molecular assays were conducted in the reference (AL-S) and lambda-cyhalothrin-resistant (AL-R) populations. Comparison of the molecular target of pyrethroid insecticides, voltage-gated sodium channel, revealed that there were no mutation sites in the resistant population, indicating target insensitivity is not responsible for increased resistance of AL-R to lambda-cyhalothrin. Furthermore, the synergism assays and the activities of detoxification enzymes were performed to determine detoxification mechanism conferring the lambda-cyhalothrin resistance. In the tested synergists, the piperonyl butoxide had the highest synergism ratio against lambda-cyhalothrin, which was up to five-fold in both populations. In addition, the result also showed that only cytochrome P450 had significantly higher O-deethylase activity with 7-ethoxycoumarin (1.78-fold) in AL-R population compared with AL-S population. Seven cytochrome P450 genes were found to be significantly overexpressed in the resistant AL-R population compared with AL-S population. Taken together, these results demonstrate that multiple over-transcribed cytochrome P450 genes would be involved in the development of lambda-cyhalothrin resistance in AL-R population.

## Introduction

The mirid bug *Apolygus lucorum* (Meyer-Dür) (Hemiptera: Miridae) had been a primary pest of cotton in northern China during the 1950’s, but its population densities always remained low owing to the frequent application of synthetic insecticides against Lepidopteran pests [1]. However, since 1997, the widespread planting of trans-*Bacillus thuringiensis* (Bt) crops has dramatically reduced insecticides use and thus spurred the emergence of mirid bugs as dominant pests in transgenic Bt cotton fields in China [2–5].

*A*. *lucorum* (Meyer-Dür) damages various crops in China, especially in region belonging to the Yellow River basin [6]. For cotton crop, both nymphs and adults feeding can induce the stunting of cotton plants and the abscission of flower buds even cotton bolls, finally leading to serious yield and quality losses [7, 8]. The sole effective management on *A*. *lucorum* relies on calendar-based application of insecticides, including pyrethroids, organophosphates, carbamates, and neonicotinoids [9]. Lambda-cyhalothrin, a type of pyrethroid insecticide, is one of the most widely used to control *A*. *lucorum*. Resistance to the pyrethroids has evolved in some heavily sprayed field populations of *A*. *lucorum* in China, for instance the resistance towards lambda-cyhalothrin (up to 29-fold) in Binzhou, Shandong Province in 2015 [10], resistance to beta-cypermethrin in the same geographical field (resistance ratio of 16-22-fold) from 2011 to 2014 [11], and an upward tendency of resistance to in other areas of Shandong Province (Juye, Huimin, and Xiajin) during 2009–2014 [12].

Pyrethroid resistance in insects is mainly attributed to mutations that reduce the binding affinity of pyrethroids to the voltage-gated sodium channel (VGSC) target [13] and also to enhanced detoxification metabolism, particularly detoxification mediated by carboxylesterase-catalyzing hydrolysis [14] and cytochrome P450-dependent oxidation [15]. The involvement of cytochrome P450 enzymes in insecticide resistance is usually evaluated with the use of synergists such as piperonyl butoxide (PBO). The resistant insects exhibited an increased susceptibility to insecticides when treated with PBO, suggesting the insecticide resistance is produced by the enhanced cytochrome P450 activity. For example, in *Lygus lineolaris* adults, application of PBO or trichloropropynyl ether increased sensitivity towards cypermethrin, indicating that the elevated P450-mediated metabolism is involved in cypermethrin resistance [16].

In pyrethroid-resistant insects, the enhanced metabolic detoxification of pyrethroids is conferred by the increased transcription of cytochrome P450 genes belonging to the CYP12, CYP6, CYP9 and CYP4 families [17–20]. In the cotton pest, Australian *Helicoverpa armigera*, over-expression of *CYP6B7* [21], *CYP9A12* and *CYP9A14* was contributed to pyrethroid resistance [22], and over-expression of *CYP337B3* conferred resistance to fenvalerate and cross-resistance to cypermethrin [23]. Pyrethroid resistance in the tarnished plant bug *L*. *lineolaris* was associated with the mutation of CYP6X1 and its mRNA up-regulation [24]. Over-expressed cytochrome P450 genes were implicated in lambda-cyhalothrin resistance in *Aphis glycines* Matsumura [25]. In arachnidan mites, P450 genes also could be associated with pyrethroid resistance. *CYP389B1* and *CYP392A26* were highly over-expressed in a fenpropathrin-resistant strain of *Tetranychus cinnabarinus* (Boisduval) [26], and *CYP391A1*, *CYP384A1*, *CYP392D11* and *CYP392A28*, are also involved in fenpropathrin resistance [27].

To understand the mechanisms of lambda-cyhalothrin resistance in the green mirid bug *A*. *lucorum*, sensitivity to synergists, enzyme activities, and the transcription levels of P450 genes were compared between the reference and lambda-cyhalothrin-resistant populations.

## Materials and methods

### Insect

Two populations of *A*. *lucorum* were studied. The reference population (AL-S, as a relative susceptibility population with heterogenous hereditary background), collected from the cotton fields of Langfang Experimental Station, Hebei Province, China (39.517966 N, 116.666811 E), has been reared in the laboratory without insecticide contact since 2008. The resistant AL-R population was derived from AL-S by selection with lambda-cyhalothrin for 11 generations at 50–70% mortality. At least 1500 adults of four days old were screened per generation by the bioassay protocol below. Per adult was treated with a droplet (0.6 μL) of lambda-cyhalothrin. After 24 h, the survivors were transferred to a clean container with fresh beans to produce offspring for the next generation. The dose of lambda-cyhalothrin was increased from 0.7 ng /adult to 55 ng /adult during the selection process.

Both populations were reared on sauteed green beans (*Phaseolus vulgaris*). The environmental conditions were set as: 26 ± 1°C, 60 ± 5% relative humidity (RH) and a 16 h:8 h light: dark photoperiod.

### Chemicals

Technical grade insecticide lambda-cyhalothrin (98% purity) was used and obtained from Jiangsu Yangnong Chemical Group Co., LTD. (Yangzhou, China). Piperonyl butoxide (PBO, 90% purity) and diethyl maleate (DEM, 90% purity) were purchased from West Chester, PA. S,S,S-Tributyltrithiophosphate (DEF), triphenyl phosphate (TPP, 90% purity), 1-chloro-2, 4-dinitrobenzene (CDNB), fast blue B salt,1-naphthyl acetate (*α*-NA), reduced glutathione (GSH), reduced nicotinamide adenine dinucleotide phosphate (NADPH) and 7-ethoxycoumarin (EC) were obtained from Sigma-Aldrich (St. Louis, MO, USA).

### Toxicity bioassay and synergism assay

The topical method was used to determine the level of resistance to lambda-cyhalothrin and the synergistic activity of detoxifying enzyme inhibitors [28]. Acetone was used as the solvent, and also as a control.

Lambda-cyhalothrin was serially diluted up to 4–7 different concentrations with 3–4 replications of each concentration. Prior to pesticide application, more than 30 *A*. *lucorum* adults of 4-day-old were anaesthetized with carbon dioxide and placed on ice for each concentration group. A droplet (0.6 μL) of lambda-cyhalothrin was applied onto the dorsum (thorax) of the adult using a semi-automatic dropper (PB-600 PAT, 3161323, USA). After treatment, ten individuals per group were placed in a plastic box with a fresh green bean pod. Mortality was calculated after 24 h.

For synergism assays, the synergists PBO, DEM, DEF, and TPP were dissolved in acetone and applied topically to the dorsal prothorax of adults of the AL-S and AL-R populations, as described above. The doses applied (30 ng of PBO, 60 ng of DEM, 60 ng of DEF or 60 ng of TPP per individual adult) caused no mortality in adults from both strains. After 1 h, the adults were treated with lambda-cyhalothrin as described for the topical bioassay.

The LD_50_ values and slopes of mortality/dose relationships were estimated by probit analysis with the computer program POLO-PC (LeOra Software, USA).

### Metabolic enzyme assays

Protein content was measured with bovine serum albumin as the standard substrate using the method of Bradford [29].

The enzyme activities of carboxylesterase (CarE), glutathione S-transferase (GST), and cytochrome-P450-dependent monooxygenase (P450) were measured using 1-naphthylacetate (*α*-NA), 1-chloro-2,4-dinitrobenzene (CDNB) and 7-ethoxycoumarin (7-EC) as substrates, respectively. The detailed procedures were described in a previous study [10].

### Amplification and sequencing of sodium channel gene and cytochrome P450 genes

Total RNA was isolated from the adults of *A*. *lucorum* (3–4 days old) using TRIzol reagent (Invitrogen, Carlsbad, CA) following the manufacturer’s specifications. First strand cDNA was synthesized from total RNA using PrimeScript™ RT reagent Kit with gDNA Eraser (Perfect Real Time) (Takara, Dalian, China). To check for target mutation, a series of cDNA fragments of the para-sodium channel gene were amplified with the primers of the previous study [10]. At least 30 adult individuals were selected for sequencing in each population.

Amplification of cytochrome P450 genes were performed with specific primers (S1 Table). The missing 3’ and 5’ ends of *CYP* genes were obtained from first strand cDNA with gene-specific primers (S1 Table) using a SMART™ RACE cDNA amplification kit (Clontech, USA). The full-length sequences of *CYPs* were then amplified using gene-specific primers (S1 Table). All PCR products were gel-purified, ligated into the pMD-18T vector (Takara, Dalian, China) and sequenced by Invitrogen (Shanghai, China).

### Real time quantitative PCR of *A*. *lucorum* P450 genes

The clean reads and computationally assembled sequences about AL-S and AL-R populations were submitted to the Sequence Read Archive (SRA) database (Accession number: SRP149628). 101 cytochrome P450 genes with the mean length of 1259 nucleotides were found via transcriptome analysis. Differential expression data between AL-S and AL-R populations revealed that 8 P450 unigenes were significantly up-regulated and 41 unigenes downregulated. The transcription profiles of 11 selected P450 genes in the AL-S and AL-R populations were determined by real-time qPCR. Specific primers (supporting information S1 Table) were designed to amplify the *A*. *lucorum* P450 and β-actin gene (reference gene). Primer pairs were optimized and tested to ensure that they yielded unique amplification products and possessed similar amplification efficiencies. The amplification efficiency of each primer pair was estimated by using the equation E = 10^−1/slope^, where the slope was derived from the plot of cycle threshold (C_t_ value) versus amount of serially diluted template cDNA.

QPCR was carried out using the ABI 7500 qPCR System with the Platinum SYBR Green qPCR SuperMix-UDG kit (Invitrogen, Carlsbad, CA). The optimized cycling conditions were 1 cycle of 2 min at 50°C, 1 cycle of 2 min at 95°C, and 40 cycles of 15 s at 95°C and 30 s at 60°C followed by a product dissociation stage (Applied Biosystems 7500). To check reproducibility, each qRT-PCR assay was performed in triplicate, and samples were repeated three times, each with a new preparation of total RNA. The relative transcript levels for each P450 gene in each population were calculated by the 2^−ΔΔCt^ method [30].

### Phylogenetic analysis

Phylogenetic analysis was conducted in order to investigate evolutionary relationships among the putative P450 proteins identified in *A*. *lucorum* and selected proteins from other insects. GenBank accession numbers of *Apis florae* P450s: CYP6J1 (XP_003690779), CYP6A2 (XP_003694559); *Anopheles funestus*: CYP6N1 (AFM08399), CYP6N2 (AFM08400); *Aedes albopictus*: CYP6N3v3 (AAF97938); *Anopheles gambiae*: CYPM3R9(AAO62002); *Aphis gossypii*: CYP6A14 (AML23850); *Acyrthosiphon pisum*: CYP6A13 (XP_016660177); *Anopheles sinensis*: CYP6P7 (KFB36103); *Culex quinquefasciatus*: CYP6B5D (XP_001867277), CYP6F1 (BAA92152); *Drosophila melanogaster*: CYP6A1 (NP_610389); *Musca domestica*: CYP6A5 (AAA82161); *Ochlerotatus sollicitans*: CYP6P10v1 (AAX48012); *Tribolium castaneum*: CYP6BK1 (EFA12637), CYP6BQ13 (EEZ99338), CYP6BQ4 (EFA02818), CYP6BQ2 (EFA02817); *Dastarcus helophoroides*: CYP6BQ21 (AGJ51944); *Lygus lineolaris*: CYP6X1v1 (AAL15173); *Locusta migratoria*: CYP6H1 (AAD39748); *Zootermopsis nevadensis*: CYP6A1 (KDR19800); *Hodotermopsis sjostedti*: CYP6AM1 (BAD84176), CYP6A13 (XP_011153933); *Liposcelis bostrychophila*: CYP6CE1 (ABN80240); *Nasonia vitripennis*: CYP6AS33 (NP_001165939); *Camponotus floridanus*: CYP6A13 (EFN69191); *Laodelphax striatella*: CYP6AX1 (AGN52754), CYP6AY3v2 (AFU86482), CYP6FJ1v2 (AFU86439); *Nilaparvata lugens*: CYP6AX1 (CAH65681). The alignment of protein sequences was performed using the multiple alignment program Clustal W in MEGA version 5.1 [31]. Tree construction was performed using the neighbor-joining method in MEGA version 5.1 [31]. The reliability of the trees was evaluated using the bootstrap procedure with 1000 replications.

### Statistical analysis

Data were expressed as Mean ± standard error (SE) deviation from triplicate experiments. The difference in expression level of each CYP gene between AL-S and AL-R population was determined by the Student’s t-test, using SPSS for Windows (SPSS, Chicago, IL, USA). One ANOVA with Tukey's Multiple Comparison Test were used for comparisons of the relative expression of *CYP6X2* by induction of lambda-cyhalothrin or not with GraphPad Prism version 5.0 (GraphPad software, San Diego, CA, USA).

## Results

### Lambda-cyhalothrin resistance dynamics

The dynamics of lambda-cyhalothrin toxicity against *A*. *lucorum* over successive lambda-cyhalothrin selected generations were determined via bioassay (Table 1). The LD_50_ value changed from 0.74 ng/adult of F0 generation to 54.09 ng/adult of F11 generation. The resistance ratio (RR) of the AL-R population to topical application of lambda-cyhalothrin increased up to 74-fold after selection for 11 generations. However, the LD_50_ value of AL-S population also increased to 9.13 ng/adult when long-term maintenance (data shown in Table 2). Hence, the net resistance ratio (RR) of the AL-R population was only 5.9-fold compared to the AL-S population.

**Table 1:** Lambda-cyhalothrin toxicity in successive generations of the laboratory selected AL-R population.

Generations	LD_50_^a^(95%CL^b^) ng /adult	Slope±SE	χ^2^ (*df*)^c^	RR^d^
F0	0.74 (0.01–4.56)	0.40±0.10	2.90(5)	1
F1	1.76 (1.25–1.87)	0.49±0.12	0.96(3)	2.4
F2	9.12 (2.12–12.72)	1.01±0.29	1.95(3)	12.3
F3	10.88 (5.69–17.10)	1.61±0.33	1.95(3)	14.7
F4	13.35 (8.21–24.78)	1.07±0.18	1.56(3)	18.0
F5	33.95 (24.84–45.21)	2.28±0.43	1.68(3)	45.9
F6	45.54 (32.85–54.41)	4.50±1.04	2.12(3)	61.5
F8	20.50 (9.69–38.53)	1.28±0.37	0.54(3)	27.7
F9	35.48 (26.13–46.93)	2.58±0.53	0.57(3)	47.9
F10	41.91 (28.79–53.71)	2.58±0.56	1.59(3)	56.6
F11	54.09 (39.57–65.54)	3.63±0.90	0.3(3)	73.1

^a^ Lethal dose (LD_50_) expressed as ng of insecticide per adult for topical assays.

^b^ Confidence limits.

^c^ Chi-square value (*χ*^*2*^) and degrees of freedom (*df*) as calculated by PoloPlus.

^d^ RR (resistance ratio) = LD_50_ of the progeny generation F_n_/LD_50_ of the parent generation F_0_.

**Table 2:** Effects of synergist on the toxicity of lambda-cyhalothrin to the reference (AL-S) and the resistant (AL-R) populations of *A*. *lucorum*.

Population	Treatment	LD_50_ 95% CL(ng/adult)	Slope±SE	χ^2^ (*df*)	SR^a^
AL-S	cyhalothrin	9.13 (3.45–16.64)	1.33±0.30	1.44(3)	1
	cyhalothrin + PBO	1.61 (0.41–2.87) *	1.57±0.44	1.41(3)	5.7
	cyhalothrin + DEM	2.88 (0.71–6.20)	1.02±0.24	1.14(3)	3.2
	cyhalothrin + DEF	4.95 (2.98–7.38)	2.47±0.42	1.80(3)	1.8
	cyhalothrin + TPP	8.21 (1.35–136)	2.00±0.70	1.59(3)	1.1
AL-R	cyhalothrin	71.54 (49.52–93.47)	2.46±0.42	0.78(3)	1
	cyhalothrin + PBO	13.93 (7.46–22.77) *	1.33±0.24	1.11(3)	5.1
	cyhalothrin + DEM	20.15 (11.18–31.62) *	1.68±0.30	1.14(3)	3.6
	cyhalothrin + DEF	27.12 (15.02–44.36) *	1.42±0.26	0.51(3)	2.6
	cyhalothrin + TPP	26.13 (14.70–43.76) *	1.28±0.23	1.72(4)	2.7

^a^ Synergistic ratio (SR) is the LD_50_ of lambda-cyhalothrin in the AL-S or AL-R populations divided by the LD_50_ of lambda-cyhalothrin in the same population also treated with a synergist.

### Synergistic effects on the toxicity of lambda-cyhalothrin

The effect of synergists on lambda-cyhalothrin toxicity in the AL-S and AL-R populations was determined by bioassays (Table 2). TPP did not have synergistic effect on lambda-cyhalothrin toxicity in the AL-S population, however, had a synergism in the AL-R population. Similar synergistic potential of PBO and DEM to lambda-cyhalothrin between the two populations were observed. The synergistic ratio of DEF to lambda-cyhalothrin was 1.8 and 2.6 for the AL-S and AL-R population, respectively.

### Detoxifying enzyme activity

**Fig 1:**
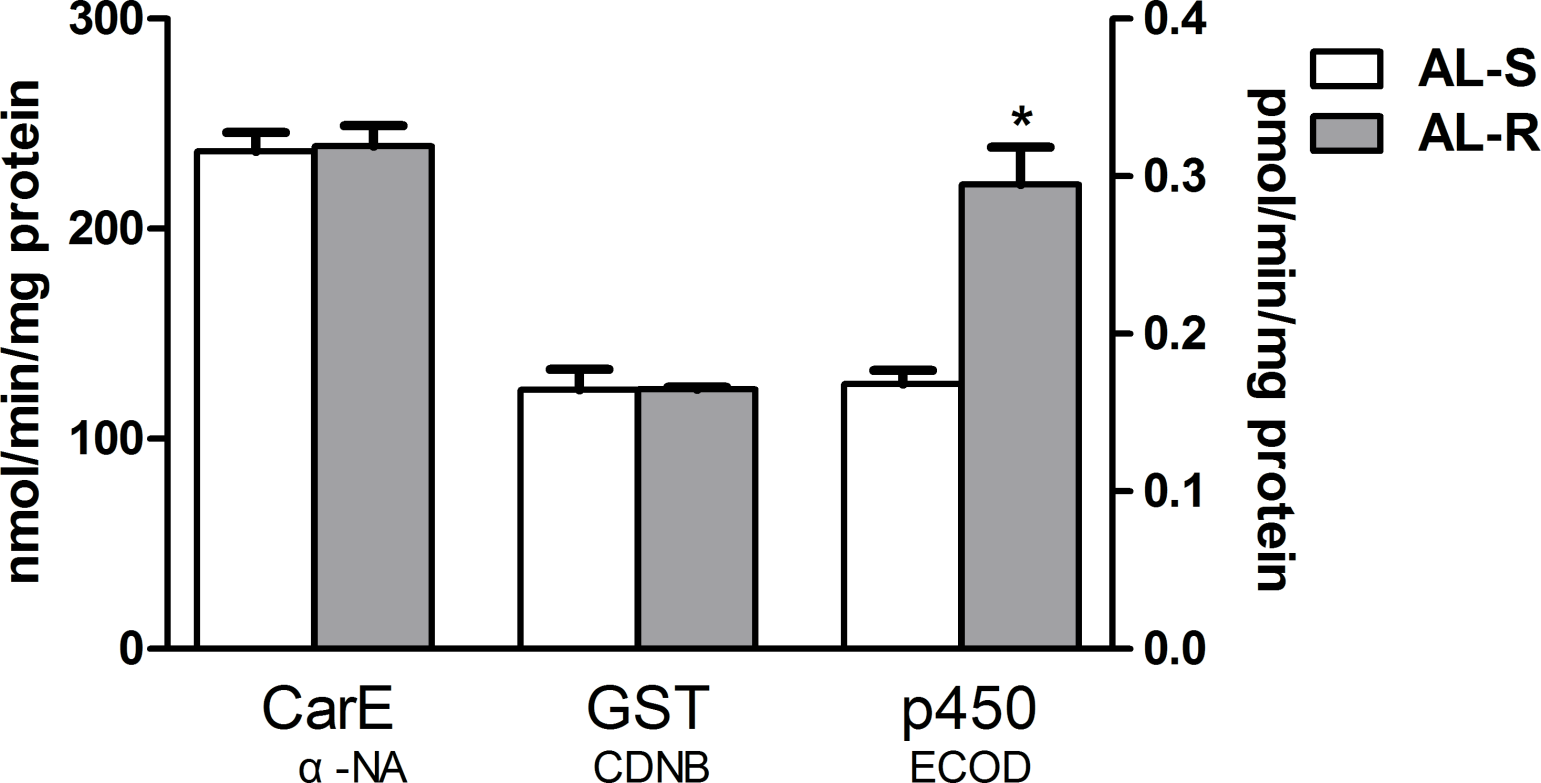
Comparison of detoxifying enzyme activity between the lambda-cyhalothrin reference and resistant populations (AL-S and AL-R). Left Y axis (mmol/min/mg protein) represents the unit of CarE and GST activities. Right Y axis (pmol/min/mg protein) represents the unit of ECOD activity.

The activities of the detoxifying enzymes CarE, GST and P450 in AL-S and AL-R populations were compared (Fig 1). The O-deethylase activity towards formation of 7-hydroxycoumarin (ECOD) of P450 was significantly higher (1.78-fold) in the AL-R population than that of the AL-S population, suggesting that lambda-cyhalothrin resistance in the AL-R population is potentially conferred by increased P450 activity.

### Comparison of para-sodium channel gene

The full complete ORF sequence of para-sodium channel was compared between AL-S and AL-R population. Through sequence comparison, no nucleotide mutation was found in the whole ORF. It was speculated that target site insensitivity didn’t account for the lambda-cyhalothrin resistance in AL-R population.

### Relative expression of CYP genes in adult mirid bugs

The relative expression of genes from the CYP4 and CYP3 clans in adults from AL-R population was determined by qPCR and compared with the expression in the AL-S population (Table 3). Among the 11 tested CYP genes, *CYP6HM1*, *CYP6HM2*, *CYP6JB1*, *CYP6JB2*, *CYP6JC1*, *CYP6X2* and *CYP395H1* had significantly higher expression levels in the AL-R population as compared to the AL-S population.

**Table 3:** Relative expression levels of *A*. *lucorum* CYP genes in AL-S and AL-R adults determined by qPCR.

*A*. *lucorum* CYP genes		Mean expression ± SE^b^		
GenBank accession number	CYP name ^a^	AL-S	AL-R	*P*-value
KY348794	*CYP4FC1*	1.009±0.003	1.024±0.285	0.655
KY348795	*CYP4EY1*	1.002±0.004	1.253±0.141	0.217
KY348797	*CYP4G114*	1.113±0.234	1.595±0.334	0.303
KY348798	*CYP6HK3*	1.253±0.141	1.021±0.129	0.293
KY264203	*CYP6X2*	1.035±0.212	20.863±3.454	0.029
KY264200	*CYP6HM1*	1.013±0.260	6.418±0.701	0.002
KY264202	*CYP6JB2*	1.032±0.136	4.706±0.684	0.006
KY264201	*CYP6JC1*	0.998±0.015	2.395±0.259	0.032
KY264199	*CYP6JB1*	1.005±0.186	6.175±0.477	0.001
KY264198	*CYP6HM2*	1.007±0.051	3.224±0.308	0.001
KY596021	*CYP395H1*	1.042±0.052	2.161±0.316	0.006

^a^ All of these gene names have been designated by David Nelson (http://drnelson.uthsc.edu/biblioB.html#6A).

^b^ The data were calculated by the 2^−ΔΔCt^ method.

**Fig 2:**
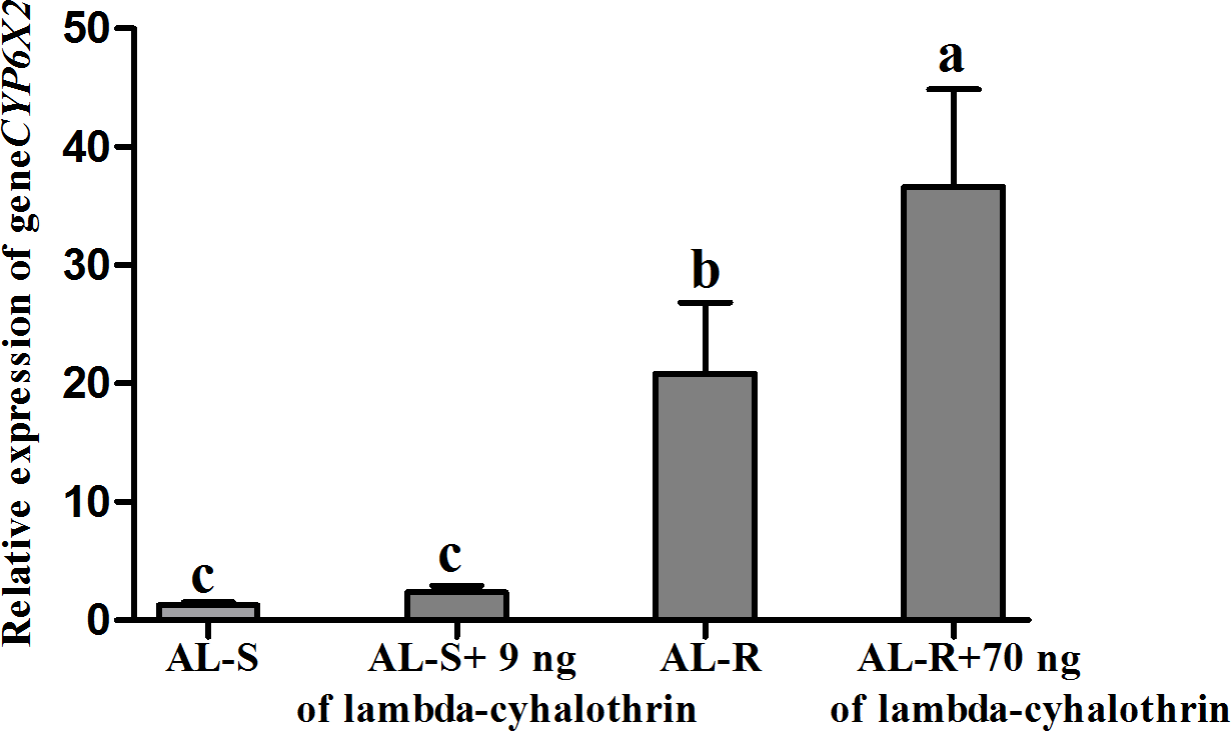
Relative expression level of the *CYP6X2* gene in adults of the control (AL-S) and lambda-cyhalothrin-resistant (AL-R) populations of *A*. *lucorum* with and without application of lambda-cyhalothrin (9 ng and 70 ng for AL-S and AL-R, respectively). Error bars indicate standard errors. Different letters indicate significant differences in relative expression level determined by Turkey’s Multiple Comparison test.

The induction of *CYP6X2* expression was also analyzed by exposing adults of the AL-S and AL-R populations to a topical droplet containing 9 and 70 ng of lambda-cyhalothrin, respectively. The result also showed that *CYP6X2* was similarly induced in both the reference (1.86-fold) and the resistant (1.54-fold) populations (Fig 2). Significant differences were again found in *CYP6X2* expression levels when comparing non-treated AL-S and AL-R populations (20.6-fold), in accordance with above mentioned results.

### Characterization of full-length CYPs

**Fig 3:**
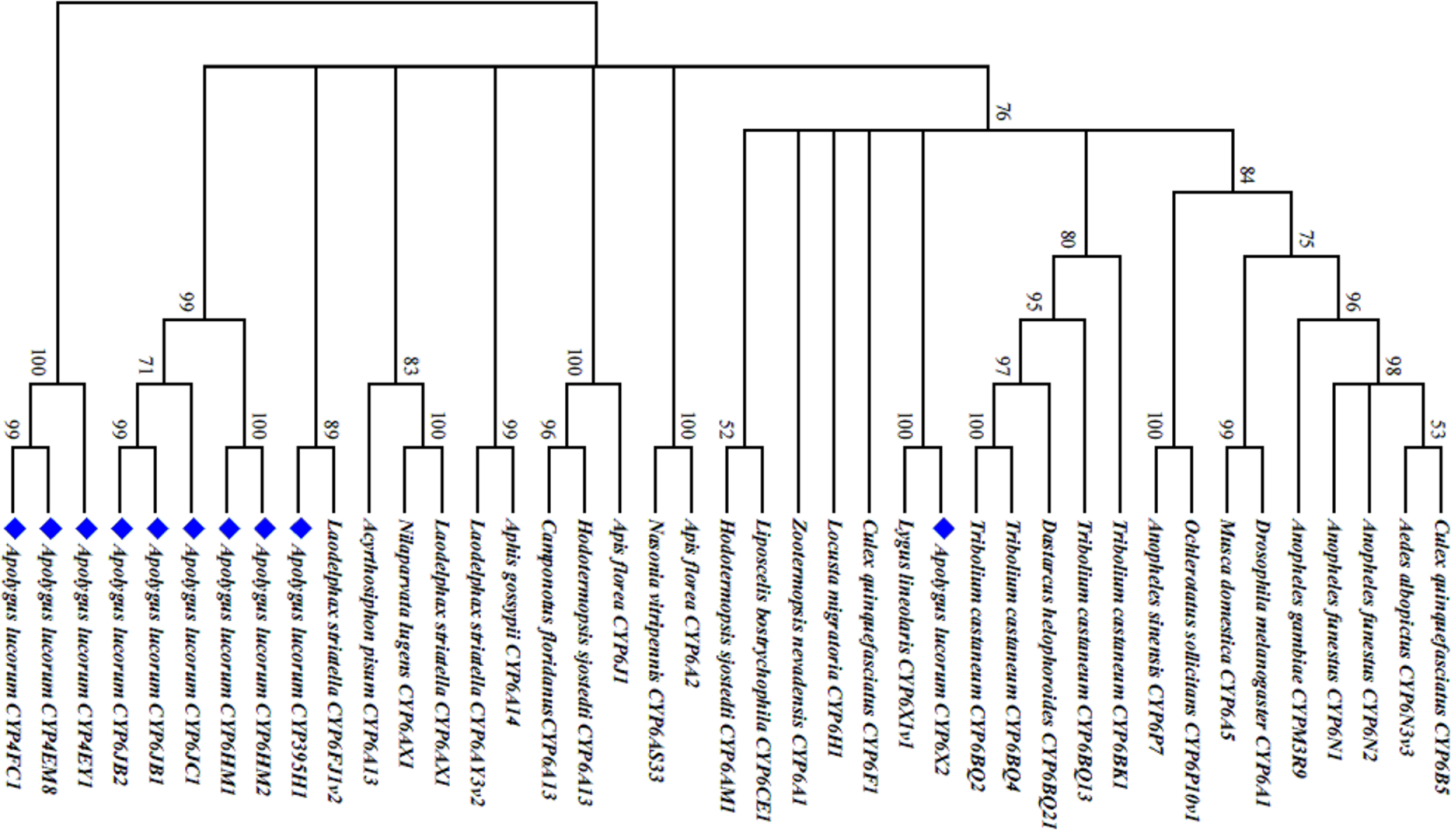
Neighbor-joining phylogeny of the deduced amino acid sequences of *Apolygus lucorum* P450s (blue diamond) and selected P450s from other insects.

The characteristic parameters of the obtained full-length *CYPs* were listed in Table 4. As shown in S1 Fig, the translated proteins of the *CYPs* possess the characteristic conserved domains including the oxygen-binding motif (helix I) ([A/G]GX[E/D]T[T/S]), the helix K motif (EXXRXXP), the heme-binding “signature” motif (PFXXGXXXCXG) and a sequence motif (PXXFXP) specific to CYP6 members. The results indicated that these *CYPs* belong to typical microsomal P450 clades. The phylogenetic tree, generated from aligned amino acid sequences of CYPs, revealed that these CYPs were closely related to those of families CYP4 and CYP6 of other invertebrate species (Fig 3).

**Table 4:** Characteristics of full-length cDNA sequences of the *CYPs* overexpressed in AL-R population.

Gene name	Size of ORF (aa)	5'UTR (bp)	3'UTR (bp)	pI	Mw (kDa)	PAS
CYP6X2	511	75	337/279	8.59	59.09	AATAAA
CYP6JB1	520	53	177	8.83	59.37	AATAAT
CYP6HM1	512	45	70	8.02	59.19	-
CYP6JC1	513	53	378	8.06	58.3	AATAAT
CYP6HM2	509	154	104	8.94	58.5	AATAAA
CYP395H1	526	-	-	7.08	60.82	-
CYP6JB2	523	-	-	8.76	59.8	-

ORF = open reading frame; UTR = untranslated region; aa = amino acids; pI = isoelectric point; Mw = molecular weight; PAS = polyadenylation signal.

## Discussion

The control of mirid bugs in Bt-transgenic cotton crop fields almost is executed by spraying chemical insecticides worldwide. Over-utilization and long-term exposure to insecticides has induced the resistance in mirid bugs. Insecticides currently approved for mirid bugs control are pyrethroids and organophosphates. For *A*. *lucorum*, some cases about the pyrethroid resistance have been reported in the Yellow river basin of China [10–12]. For the tarnished plant bug *L*. *lineolaris*, the resistance to pyrethroid insecticides occurred in the mid-south cotton-growing areas of the USA [24]. Hence, it is necessary to elucidate the potential reason for the development of pyrethroids resistance in mirid bugs.

Pyrethroid resistance mechanisms were usually complex, which mainly based on the pest, field environment, and insecticide application. A previous study of pyrethroid resistance in *A*. *lucorum* found an association between target site insensitivity due to a substitution (L1015F) and pyrethroid resistance [10]. Another study involving *Lygus* species found that resistance was correlated with increased activity of P450 detoxifying enzymes [16]. In the present study, no mutation was found in the para-sodium channel of AL-R population, suggesting that target insensitivity is unlikely to be involved in lambda-cyhalothrin resistance development. It was common that one or multiple mechanisms may be lying in different pyrethroid resistant populations of the same insect species because of the different insecticide selection pressure.

Xu et al found that the synergism of PBO to lambda-cyhalothrin was obvious with the synergism ratio up to 7.2 compared with the other three types of insecticides in *A*. *lucorum* [32], which was consistent with our significant synergistic effects of PBO to lambda-cyhalothrin in both AL-S and AL-R populations. The explanation for the obvious synergism of PBO to lambda-cyhalothrin in AL-S population was that the susceptibility of AL-S population to lambda-cyhalothrin was distinctly decreased during the long term rearing with the food *P*. *vulgaris* containing pesticide residue. The further biochemical assays confirmed indeed that the resistant individuals had higher level of P450 activities compared with reference individuals. These evidences pointed to a P450-mediated metabolic resistance mechanism involved in lambda-cyhalothrin resistance of AL-R population. Nevertheless, other metabolic mechanisms, such as glutathione S-transferase, and esterase mediated metabolisms, should not be excluded, because synergists DEM, DEF, and TPP also increased the toxicity of lambda-cyhalothrin in AL-R population. This phenomenon was similar with the enhanced detoxification rather than target insensitivity mechanism found in deltamethrin resistant *L*. *striatellus* [33].

In the Order Hemiptera, a variety of studies have documented pyrethroid resistance associated with P450s [24, 25, 33, 34], esterases [33, 35–37] and glutathione S-transferases [38, 39]. Based on previous transcriptome analysis, 49 P450 unigenes were differentially expressed between the resistant and reference populations, including 8 P450 unigenes upregulated and 41 unigenes downregulated. The expression patterns of 8 upregulated P450 unigenes and 3 insecticide resistance related P450 genes were further analyzed via qPCR. Our results showed that *CYP6HM1*, *CYP6HM2*, *CYP6JB1*, *CYP6JB2*, *CYP6JC1*, *CYP6X2*, and *CYP395H1* are more highly increased in the AL-R population than the AL-S population (Table 3). All of the seven elevated P450s belong to the CYP6 family. CYP6 family was more frequently found involving in insecticide resistance than any other P450 family [40]. For example, CYP6X1 in *L*. *lineolaris* was associated with pyrethroid resistance [24], while our CYP6X2 was highly similar to CYP6X1 of *L*. *lineolaris* (up to 82% amino acid sequence identity). CYP6AY3v2 in *Laodelphax striatellus* (Fallén) associated with deltamethrin resistance [33], while our CYP395H1 showed 34% similarity with CYP6AY3v2 of *L*. *striatellus*. CYP6F1 in *C*. *quinquefasciatus* resistant to pyrethroids [41]. CYP6A51 in *Ceratitis capitate* was resistant to lambda-cyhalothrin [42]. Moreover, inducibility by insecticide is a typical characteristic of some P450 genes involved in insecticide resistance [43–46]. In our case, the expression of *CYP6X2* was also induced in both the AL-R (1.54-fold) and AL-S populations (1.86-fold) when adults were treated with a dose of lambda-cyhalothrin equivalent to their corresponding LD_50_ values. Therefore, we hypothesize that *CYP6X2* gene may play a relevant role in the resistance of the AL-R population to lambda-cyhalothrin by over-expression of a lambda-cyhalothrin-inducible gene. However, the over-expression of P450 genes does not necessarily correlate with insecticide resistance [47]. Further works are needed to demonstrate unequivocally the role of CYP6X2 in resistance to lambda-cyhalothrin, including the metabolism of lambda-cyhalothrin by recombinant CYP protein.

Elevated expression of P450 genes in resistant insects may be achieved through increased transcription by mutations/insertions/deletions in cis-acting promoter sequences [17]. There has been a report of the insertion of a 15 bp fragment close to the transcription start site (−15 to −29) in the 5’-flanking region of the *CYP6D1* gene in permethrin-resistant strains of *M*. *domestica*, which was absent in susceptible strains [48]. Therefore, comparison of the 5’UTR and promoter sequences was necessary for identifying regions responsible for the up-regulation of *CYP6X2*.

## Conclusions

The metabolic resistance mediated by P450 appears to be the main resistance mechanism in the resistant AL-R population. Although, our data could not firmly conclude that up-regulation of the seven identified detoxification genes are associated with the observed lambda-cyhalothrin resistance, it certainly provides a solid basis for future functional studies of encoded proteins and resistance mechanism confirmation.

## Supporting information

S1 Fig Full-length mRNA and amino acid sequence of CYPs *A*. *lucorum*. Conserved amino acid domains common to cytochrome P450s are highlighted as follows: the helix I, helix K, PERF and heme-binding motif is shaded in yellow, blue, grey and purple respectively. A CYP6X2 B CYP6JB1 C CYP6HM1 D CYP6JC1 E CYP6HM2 F CYP6JB2 G CYP395H1.
(RAR)

S1 Table Sequences of primers used in this study. (DOCX)
