# Supplementary material for: Overexpression of cytochrome P450s in a lambda-cyhalothrin resistant population of Apolygus lucorum (Meyer-Dür)
Source: PLoS One. 2018 Jun 27;13(6):e0198671. doi: 10.1371/journal.pone.0198671 (PMC6021084; doi:10.1371/journal.pone.0198671)
Supplement: S1 Table — (DOCX) [file pone.0198671.s002.docx]

**Table S1.** Sequences of primers used in this study.

| Primer name | Sequence (5’-3’) | Target |
| --- | --- | --- |
| CYP4FC1-FP | TCGTTCAGAAGATGTTGGAG | qPCR for *CYP4FC1* |
| CYP4FC1-RP | GTTCAAAGTCACACCCATTG |  |
| CYP4EY1-FP | GAGGTAATCCTGACAAGCAC | qPCR for *CYP4EY1* |
| CYP4EY1-RP | GCTGAGTAGAAGTCCATCAT |  |
| CYP4G114-FP | ACCAAGTCAACACCATTATG | qPCR for *CYP4G114* |
| CYP4G114-RP | GATCTCTTCAAGGACCTTCT |  |
| CYP6X2-FP | CGTCTTCAGTCCAGAGCTAC | qPCR for *CYP6X2* |
| CYP6X2-RP | ACCCGAGTCAGAGTGTGTAG |  |
| CYP6HK3-FP | ACCATTTCGTCGACAGAGGT | qPCR for *CYP6HK3* |
| CYP6HK3-RP | TCCGTCGTAAATCTGGCTGT |  |
| CYP6JB1-FP | ACCCAGTTATAACGCGATGC | qPCR for *CYP6JB1* |
| CYP6JB1-RP | TGACGTCACTACCTGGAATG |  |
| CYP6JC1-FP | CAACCATCAGTTACGCCATG | qPCR for *CYP6JC1* |
| CYP6JC1-RP | TTCGTCACCTTCCTCGTCAG |  |
| CYP6HM1-FP | TCGAAGCAGTAGGTGAGATG | qPCR for *CYP6HM1* |
| CYP6HM1-RP | CGGTAGAACAACATCAGTAC |  |
| CYP6HM2-FP | AATACCGGGTACTAATGTCG | qPCR for *CYP6HM2* |
| CYP6HM2-RP | TTCAACGCTGGGTCGAATCT |  |
| CYP6JB2-FP | AGCTATGTCTGGCGATGATG | qPCR for *CYP6JB2* |
| CYP6JB2-RP | TAGCCACCCAAATTCCACCT |  |
| CYP395H1-FP | CCAGCTGTTCGTGACGTTGC | qPCR for *CYP395H1* |
| CYP395H1-RP | GCGACCAGAAATATGAAGGC |  |
| β-actin-FP | ACCTGTACGCCAACACCGT | qPCR for reference gene β-actin |
| β-actin-RP | TGGAGAGAGAGGCGAGGAT |  |
| FP | CTGATGTACAGTCTATTCGT | *CYP6X2* (transcriptome) |
| RP | CTTTCTGTGGATCAGCGATC |  |
| 5’RP | GATGCACGTCATGAACATAT | 5’RACE |
| 5’RNP | GAAGGACTCGTCCGGGTTAT |  |
| 3’FP | TGTTGGAAGGAGACCTGATC | 3’RACE |
| 3’FNP | TGTTCATGACGTGCATCAAT |  |
| 6X2-FP | TGTACAGGCACAATGATTTAT | *CYP6X2* (ORF) |
| 6X2-RP | TCACACACTTTCTAACTTGAG |  |
